# Does Antibiotic Treatment Before Bone Biopsy Affects the Identification of Bacterial Pathogens From Bone Culture in Diabetic Foot Osteomyelitis? A Systematic Review and Meta Analysis

**DOI:** 10.1111/wrr.70171

**Published:** 2026-05-15

**Authors:** Lawrence A. Lavery, Arthur N. Tarricone, Andrew William Ernest Madsen, Nitish Thirugnanasambandam, Allen Gee, Dane K. Wukich, Edgar J. G. Peters

**Affiliations:** ^1^ Department of Orthopedic Surgery The University of Texas Health Science Center San Antonio Texas USA; ^2^ Department of Medicine Long School of Medicine at University of Texas San Antonion San Antonio Texas USA; ^3^ Department of Medicine Mount Sinai Morningside Hospital New York New York USA; ^4^ Department of Prosthetics and Orthotics UT Southwestern School of Health Professions, Dallas Texas and Department of Orthopaedic Surgery UT Southwestern Medical Center Dallas Texas USA; ^5^ Department of Internal Medicine VU University Medical Center Amsterdam the Netherlands

**Keywords:** antibiotics, diabetes, foot ulcer, infection, osteomyelitis

## Abstract

Our aim was to evaluate the effect of pre‐biopsy antibiotics and bacterial pathogen recovery after bone biopsy in patients suspected of having diabetic foot osteomyelitis. A meta‐analysis following PRISMA guidelines was conducted. Pubmed, EMBASE, and Cochrane Library were searched for comparative studies reporting positive culture outcomes stratified by antibiotic exposure prior to bone biopsy. Eligible studies included adult patients undergoing bone biopsy with extractable data on pre‐biopsy antibiotic use and culture positivity. Pooled odds ratios were calculated using a random‐effects model with the Mantel–Haenszel method. Statistical heterogeneity was assessed using the *I* ^2^ statistic and Cochran's *Q* test. Four studies met inclusion criteria. The cumulative findings showed an odds ratio of 0.78, CI [0.31, 1.98], *p* = 0.60, suggesting no significant difference in bacterial culture yield between groups that had antibiotic exposure and patients naïve to antibiotics before biopsy. Heterogeneity: *τ* ^2^ (REML^b^) = 0.42; *χ* ^2^= 6.11, *p* = 0.11; *I* ^2^ = 50%. Withholding antibiotics before bone biopsy in DFO patients does not appear to affect bacterial culture yield.

## 1 Introduction

Withholding antibiotics prior to obtaining bone cultures to treat diabetic foot osteomyelitis (DFO) is a controversial topic. It is part of the belief system taught in many medical schools and residency programs. Withholding antibiotics prior to bone culture is endorsed by consensus guidelines, even though the evidence does not strongly support this position. The IDSA and IWGDF recommend withholding antibiotics prior to bone biopsy when it is safe [1, 2]; despite evidence that pre‐biopsy treatment does not impact culture results in vertebral and diabetic foot osteomyelitis [3, 4].

The overwhelming majority of DFO cases occur from direct inoculation from a contiguous diabetic foot ulcer, leading to infection of the bone or septic arthritis [5]. Surrounding soft tissue infection and abscess are very common, so in most cases antibiotics are initiated to treat the soft tissue infection while the presence of osteomyelitis is being investigated. Recommendations for withholding antibiotics are based on the concern that pre‐biopsy antibiotics could negatively impact bacterial pathogen yield or result in alteration of the type of pathogens recovered. In clinical terms, false‐negative cultures could occur that would impact selection of the proper antibiotic. Another question to ask is if withholding antibiotics is important, how long should antibiotics be withheld prior to biopsy. Guideline recommendations vary from 3 days to preferably 2 weeks. To the best of our knowledge, we have not been able to identify literature that supports these time frames [1]. The objective of this meta‐analysis is to evaluate the existing evidence for withholding antibiotics in people suspected of having DFO.

## 2 Methods

**FIGURE 1:**
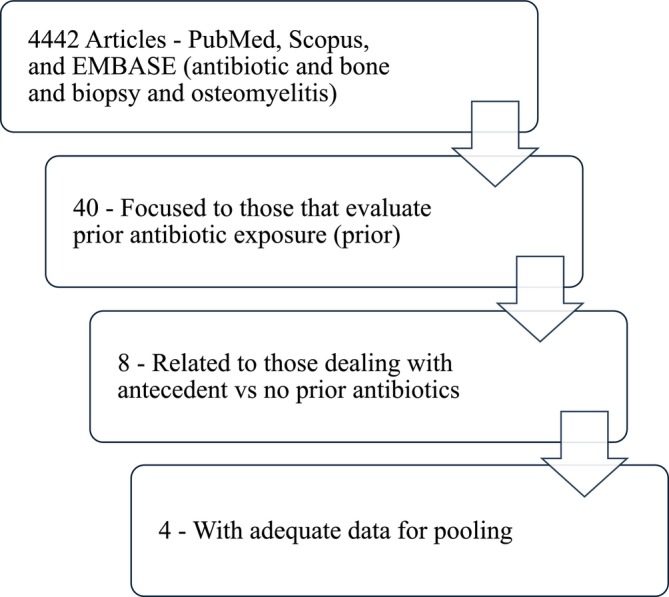
Flow chart of study selection.

We performed a literature search using 4 databases: PubMed, Web of Science, Embase/Medline, and Cochrane Central Register of Controlled Trials on November 15, 2025. We used medical subject (MeSH) and Boolean operators in the search strategy (Figure 1 Flow Chart of literature search). This meta‐analysis was conducted in accordance with the preferred reporting items for systematic reviews and meta‐analyses (PRISMA) Checklist [6]. We registered the search strategy with Research Registry under the protocol number 2076.

Duplicate manuscripts were removed after all searches were populated. The remaining papers were screened by title and abstract. We included subjects with diabetic foot infection that were suspected of having osteomyelitis that required bone biopsy to identify the presence of osteomyelitis and the bacterial pathogens that were obtained from bone culture. Consistent with the research aim, patients that received antibiotics were compared to patients that did not receive antibiotics before bone biopsy. Case reports, basic science research, non‐human populations, non‐English translatable studies, systematic reviews, meta‐analyses, and patient populations without diabetes or osteomyelitis were excluded. Screening of journal articles was conducted independently by two reviewers (N.T. and A.M.). After reading the available literature, two authors independently reviewed the variables and outcomes for each paper. Disagreements were resolved and by consensus of the reviewers and through a third reviewer (A.G.).

The primary outcome was the proportion of bone biopsies that had positive cultures. This outcome was selected to evaluate the potential impact of pre‐biopsy antibiotic exposure on culture yield, which is the primary concern underlying current guideline recommendations to withhold antibiotics. The primary analysis of this study compared people who received antibiotics before bone culture and people who did not receive antibiotics.

Statistical analysis was performed using STATA BE17 and Cochrane Review Manager (RevMan) to compute odds ratios (OR) with 95% confidence intervals (95% CI) and publication bias using Egger's plots. Higgin's and Tompson's *I* ^2^ statistic was used to quantify heterogeneity [7]. Since studies had moderate and high heterogeneity, a random‐effects model was used to account for variability among studies. Forest plots and computed effect sizes using RevMan were created. Continuous variables were reported as means and standard deviation, and categorical variables as frequency (%). An alpha of 0.80 and *p* ≤ 0.05 were selected. Cochran's *Q*, *I* ^2^, *τ* ^2^, and *τ* evaluated study heterogeneity. Risk of bias was assessed using the ROBINS‐I tool for non‐randomised studies.

## 3 Results

### 3.1 Study Selection

A total of 4442 articles were identified through a thorough database search in PubMed, Embase/Medline, Web of Science, and the Cochrane Central Register of Controlled Trials. After removal of duplicate manuscripts, titles and abstracts were screened according to predefined inclusion and exclusion criteria. Studies were exluded if they were case reports, review articles, non‐human studies, non‐diabetic populations, or did not report bone culture outcomes stratified by antibiotic exposure prior to bone biopsy. Following full‐text review, four studies were included in our analysis. Two of the authors independently evaluated the data (N.T. and A.M.).

### 3.2 Study Characteristics

The four included studies comprised a total of 623 patients with suspected diabetic foot osteomyelitis who underwent bone biopsy for diagnostic confirmation and microbiological culture. Two studies were prospective observational studies, and two were retrospective cohort studies. The studies were conducted in the United States, the Netherlands, France and Spain, reflecting diverse clinical practice settings. Bone biopsy techniques included percutaneous bone biopsy, open surgical biopsy, intraoperative bone sampling, and bone biopsy obtained through ulcer beds following debridement, reflecting variability in sampling approaches across studies. Diagnostic reference standards for osteomyelitis varied across studies and included combinations of clinical findings, imaging, probe to bone testing, laboratory markers and histopathological confirmation (Table 1).

**TABLE 1:** Study characteristics.

Author (Country)	Design study	Method	Pre‐biopsy antibiotics vs. no antibiotics	Antibiotic effect	Reference standard
Gramberg et al. [8] (Netherlands)	Prospective *N* = 64	Percutaneous bone and ulcer bed	29 (83%) vs. 35 (80%)	No effect on culture yield.	Clinical signs, imaging, ESR ≥ 70, probe‐to‐bone, biopsy histology
Lavery et al. [3] (United States)	Retrospective *N* = 114	Intra op bone biopsies	88 (79.5%) vs. 26 (84.6%)	No effect on Culture yield.	Histology
Lesens et al. [9] (France)	Retrospective *N* = 80	Per wound bone biopsy after surgical debridement	42 (95.2%) vs. 38 (100%)	No effect on culture yield.	Clinical signs, Positive probe to bone, and radiological changes
Cecilia‐Matilla et al. [10] (Spain)	Observational Prospective study *N* = 165	Open	137 (87.8%) vs. 28 (60.7%)	No effect on Culture yield	Clinical signs, probe to bone, and radiologic changes

### 3.3 Baseline Demographics and Clinical Characteristics

Across the included studies, all subjects had diabetes mellitus by study design. Age was reported either as median with minimum to maximum values or as mean ± standard deviation, depending on the original study. Median ages generally fell within the 6th to 7th decade of life, with reported age ranges extending from approximately 40 to over 90 years across studies. Male patients predominated in all cohorts, accounting for approximately 70%–75% of the participants where sex distribution was reported. Duration of diabetes was inconsistently reported and, in studies providing these data, the median duration of diabetes ranged from 4 to 55 years. Markers of vascular disease were heterogeneously reported. Absent pedal pulses, defined as the absence of one or more pedal pulsations on physical examination, were reported in one cohort and represent a clinical examination ending rather than a diagnostic criterion for peripheral artery disease. Peripheral artery disease, defined according to study‐specific diagnostic criteria, was reported in a subset of studies. Chronic kidney disease was also variably reported, with prevalence ranging from approximately 20%–35% among cohorts where data were available.

### 3.4 Primary Outcome: Bone Culture Yield

All four studies reported the proportion of positive bone cultures in patients who received antibiotics prior to bone biopsy compared with those who were antibiotic naive. Across studies, bone culture positivity rates remained high in both groups, ranging from approximately 61% to 100% in patients not exposed to antibiotics and approximately 80%–95% in patients who received antibiotics prior to biopsy. None of the individual studies demonstrated a statistically significant reduction in bacterial culture yield associated with pre‐biopsy antibiotic exposure.

**FIGURE 2:**
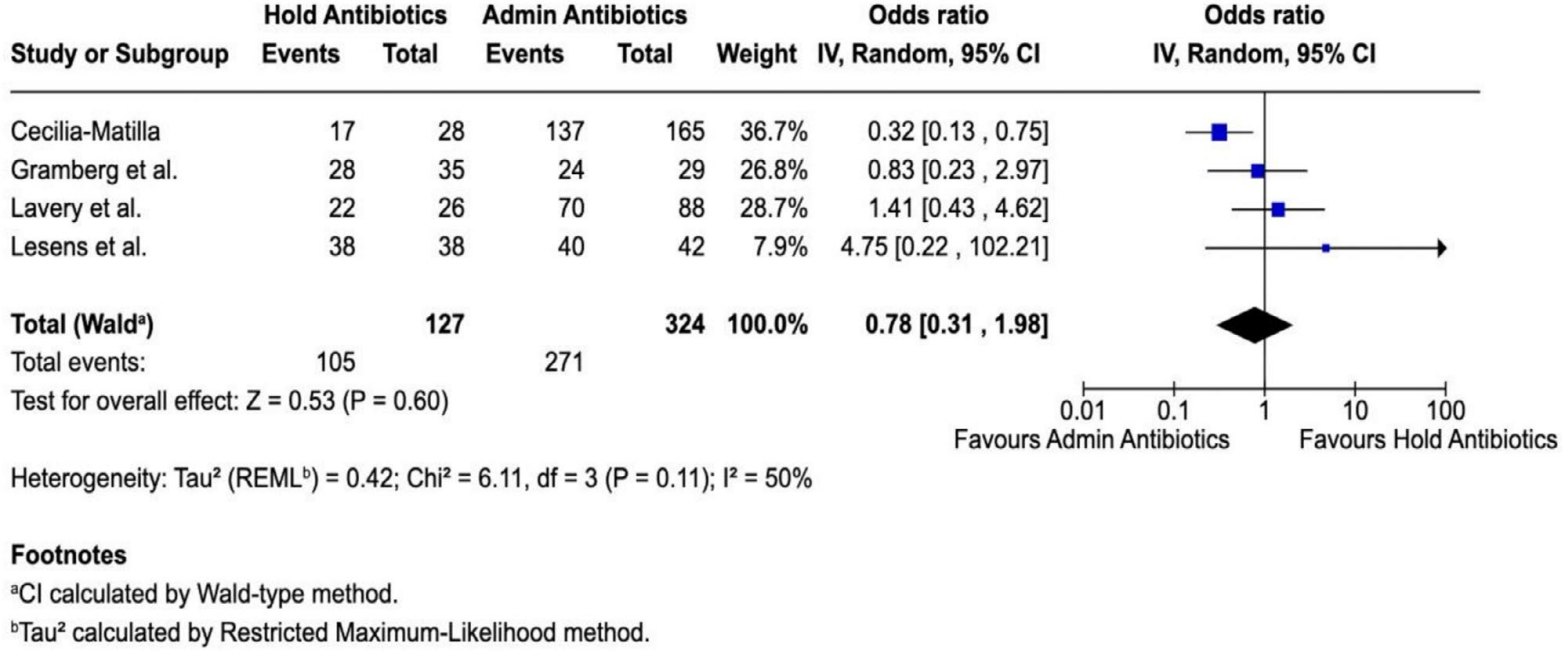
Positive culture rate between holding and administering antibiotics.

A random‐effects meta analysis using the inverse variance method was performed to account for the inter‐study variability. The Forest plot (Figure 2) depicts a graphical representation of the relation of the sample size, confidence interval, and relative study weight as represented by the area of the circles in the figures. The odds ratio (OR) for withholding antibiotics in all studies was 0.78 (95% CI, 0.31–1.98, *p* = 0.60). There was no difference in antibiotic yield in people that were treated with antibiotics before a bone biopsy and in people that were not exposed to antibiotics before the bone biopsy.

For all studies, a *Q* value of 6.11 was determined with a *P*_*Q*_ < 0.0001, indicating that moderate heterogeneity exists among the studies. The *I* ^2^ (proportion of observed variance) was found to be 50%, which suggested the populations are not similar. The *τ* ^2^ and *τ* were 0.42 and 0.65, respectively. Given the heterogeneity and results of the Forest plot, the combined data failed to identify a significant effect of preoperative antibiotics on the culture yield for bone biopsy to diagnose osteomyelitis.

## 4 Discussion

The results of this meta‐analysis did not detect a significant difference in bacterial pathogens recovered in patients that are treated with antibiotics before bone biopsy and patients that are not treated with antibiotics. Two studies found higher bone biopsy culture yields in patients who did not receive pre‐biopsy antibiotics compared to patients who received pre‐biopsy antibiotics [8, 10]. In contrast, in the study by Cecilia‐Matilla et al., higher culture positivity in patients who received antibiotics may reflect greater infection severity and earlier treatment initiation, suggesting that disease severity may act as a confounder in observational cohorts.

Many practice patterns are still driven by handed down (maybe legacy) traditions that have been based on a hypothetical rationale rather than evidence. Guidelines from the IDSA and International Working Group on the Diabetic Foot [1], and the Société de Pathologie Infectieuse de Langue Française [11] endorse withholding antibiotics for 2 weeks before obtaining bone cultures in DFO. The same guidance to withhold antibiotics, whenever possible, is recommended for vertebral osteomyelitis [12] and paediatric osteomyelitis [13] by IDSA clinical practice guidelines, to avoid false positives. There is a growing body of evidence involving all of these areas. It is important to note that the pathoetiology of vertebral osteomyelitis and paediatric osteomyelitis is quite different than DFO as the former two infections are overwhelmingly hematogenously seeded.

This meta‐analysis dichotomizes pre‐biopsy treatment with antibiotics. There was insufficient data to evaluate the duration, timing, or type of antibiotic exposure before biopsy for DFO from the meta‐analysis. Young and colleagues evaluated the duration of antibiotics in 381 patients admitted to hospital for soft tissue or bone infections and found that longer antibiotic exposure before culture was associated with lower rates of streptococcus and anaerobes, but had no effect on *Staphylococcus aureus* and gram negative pathogens [14]. There is also a dearth of information about clinical outcomes as well. Muri and colleagues reported the results of a retrospective study of 1235 patients admitted to hospital with moderate to severe diabetic foot infection (DFI) and compared outcomes of patients that received preoperative antibiotics (mean duration: 6.4 weeks median duration: 13 days) and people that did not receive antibiotics before culture. The results of multivariate logistic regression showed that the duration of presurgical antibiotic treatment was not related to clinical failure or microbiological recurrence [15].

Guidelines for diabetic foot, vertebral, and paediatric osteomyelitis as well as prosthetic joint infections all recommend that antibiotics should be withheld unless there is an acute need for antibiotics such as sepsis, cellulitis, deep abscess, or ascending infection. People with diabetes and foot infections represent a distinct clinical subset compared to other forms of osteomyelitis. Diabetic foot osteomyelitis most commonly occurs through contiguous spread from a neuropathic ulceration to the joint capsule or bone, rather than from a hematogenous source, and is frequently associated with surrounding soft tissue infection [16]. In contrast, vertebral and paediatric osteomyelitis are more commonly hematogenously seeded infections [12, 13]. In addition, people with diabetes often have impaired immune function, including abnormalities in cytokine production, neutrophil function, phagocytosis, chemotaxis, and oxidative burst activity [17, 18, 19]. These pathophysiological differences, along with the presence of peripheral ischemia, may result in a higher local bacterial burden that is less susceptible to short‐term antibiotic suppression, which may help explain the similar culture yields observed despite pre‐biopsy antibiotic exposure. For instance, Cecilia‐Matilla and colleagues evaluated 165 people with DFO and reported that 32.1% did not manifest clinical signs of infection [10]. Should we rely on the traditional signs and symptoms of infections, that all guidance documents support, to start or withhold antibiotics in a severely impaired host?

There are important limitations in this meta‐analysis. Only four studies were included but several other papers were identified that specifically addressed the topic of withholding antibiotics in people with diabetic foot infections, however, they did not report bacterial pathogen yield in the two groups [14, 15, 20]. The time period antibiotics were withheld was not clearly stated in any of the papers and the duration of antibiotics given in the pre‐biopsy treatment groups was inconsistently provided. The variability in biopsy techniques across studies, including sampling through ulcer beds, may have influenced culture positivity rates due to potential contamination. However, the absence of a significant difference across studies using different sampling approaches suggests that antibiotic exposure may not be the primary determinant of culture yield. In addition, the included studies are observational and demonstrate moderate heterogeneity despite the use of a random‐effects model. While the pooled analysis of 623 patients reflect the current available evidence and shows no significant difference in culture yield, these findings cannot be applied at the individual patient level, and clinical decision‐making should remain guided by patient‐specific factors.

## 5 Conclusion

The results of this meta‐analysis did not detect a significant difference in bacterial yield in patients who received pre‐biopsy antibiotics and those who did not; however, the available evidence is limited, and clinically meaningful effects cannot be excluded. In future studies, we need to evaluate the duration and type of pre‐biopsy antibiotics and their effect on the prevalence and type of bacterial pathogens as well as clinical outcomes.

## Conflicts of Interest

The authors declare no conflicts of interest.

## Supporting information


**Supporting Information: S1** Search strategy.
**Supporting Information: S2** Risk of bias.
**Supporting Information: S3** Funnel plot and eggers test.

## Data Availability

The data that support the findings of this study are available on request from the corresponding author. The data are not publicly available due to privacy or ethical restrictions.
