# Supplementary material for: Does Antibiotic Treatment Before Bone Biopsy Affects the Identification of Bacterial Pathogens From Bone Culture in Diabetic Foot Osteomyelitis? A Systematic Review and Meta Analysis
Source: Wound Repair Regen. 2026 May 15;34:e70171. doi: 10.1111/wrr.70171 (PMC13179428; doi:10.1111/wrr.70171)
Supplement: Supplementary file 1 — Supporting Information: S1 Search strategy. Supporting Information: S2 Risk of bias. Supporting Information: S3 Funnel plot and eggers test. [file WRR-34-0-s001.docx]

**Supplementary 1: Search Strategy**

PubMed

Search: (Antibiotic) AND (Bone) AND (Biopsy) AND (Osteomyelitis) ("anti bacterial agents"[Pharmacological Action] OR "anti bacterial agents"[Supplementary Concept] OR "anti bacterial agents"[All Fields] OR "antibiotic"[All Fields] OR "anti bacterial agents"[MeSH Terms] OR ("anti bacterial"[All Fields] AND "agents"[All Fields]) OR "antibiotics"[All Fields] OR "antibiotic s"[All Fields] OR "antibiotical"[All Fields]) AND ("bone and bones"[MeSH Terms] OR ("bone"[All Fields] AND "bones"[All Fields]) OR "bone and bones"[All Fields] OR "bone"[All Fields]) AND ("biopsie"[All Fields] OR "biopsy"[MeSH Terms] OR "biopsy"[All Fields] OR "biopsied"[All Fields] OR "biopsies"[All Fields] OR "biopsy s"[All Fields] OR "biopsying"[All Fields] OR "biopsys"[All Fields] OR "pathology"[MeSH Subheading] OR "pathology"[All Fields]) AND ("osteomyelities"[All Fields] OR "osteomyelitis"[MeSH Terms] OR "osteomyelitis"[All Fields] OR "osteomyelitides"[All Fields])

1764 Results

Search: (Bone Biopsy) AND (Osteomyelitis) AND (Antibiotic) Filters: Full text, Clinical Trial, Randomized Controlled Trial (("bone and bones"[MeSH Terms] OR ("bone"[All Fields] AND "bones"[All Fields]) OR "bone and bones"[All Fields] OR "bone"[All Fields]) AND ("biopsie"[All Fields] OR "biopsy"[MeSH Terms] OR "biopsy"[All Fields] OR "biopsied"[All Fields] OR "biopsies"[All Fields] OR "biopsy s"[All Fields] OR "biopsying"[All Fields] OR "biopsys"[All Fields] OR "pathology"[MeSH Subheading] OR "pathology"[All Fields]) AND ("osteomyelities"[All Fields] OR "osteomyelitis"[MeSH Terms] OR "osteomyelitis"[All Fields] OR "osteomyelitides"[All Fields]) AND ("anti bacterial agents"[Pharmacological Action] OR "anti bacterial agents"[Supplementary Concept] OR "anti bacterial agents"[All Fields] OR "antibiotic"[All Fields] OR "anti bacterial agents"[MeSH Terms] OR ("anti bacterial"[All Fields] AND "agents"[All Fields]) OR "antibiotics"[All Fields] OR "antibiotic s"[All Fields] OR "antibiotical"[All Fields])) AND ((clinicaltrial[Filter] OR randomizedcontrolledtrial[Filter]) AND (fft[Filter]))

16 Results

Embase/Medline

| Search # | Term | Results |
| --- | --- | --- |
| 1 | Antibiotic | 18889911 |
| 2 | Bone Biopsy | 29578 |
| 3 | Osteomyelitis | 165704 |
| 4 | 1 AND 2 | 3723 |
| 5 | 2 AND 3 | 5587 |
| 6 | 1 AND 2 AND 3 | 2678 |

Web Of Science

0 Results

Cochrane Central Register of Controlled Trials

0 Results

**Supplementary 2: Risk of Bias**


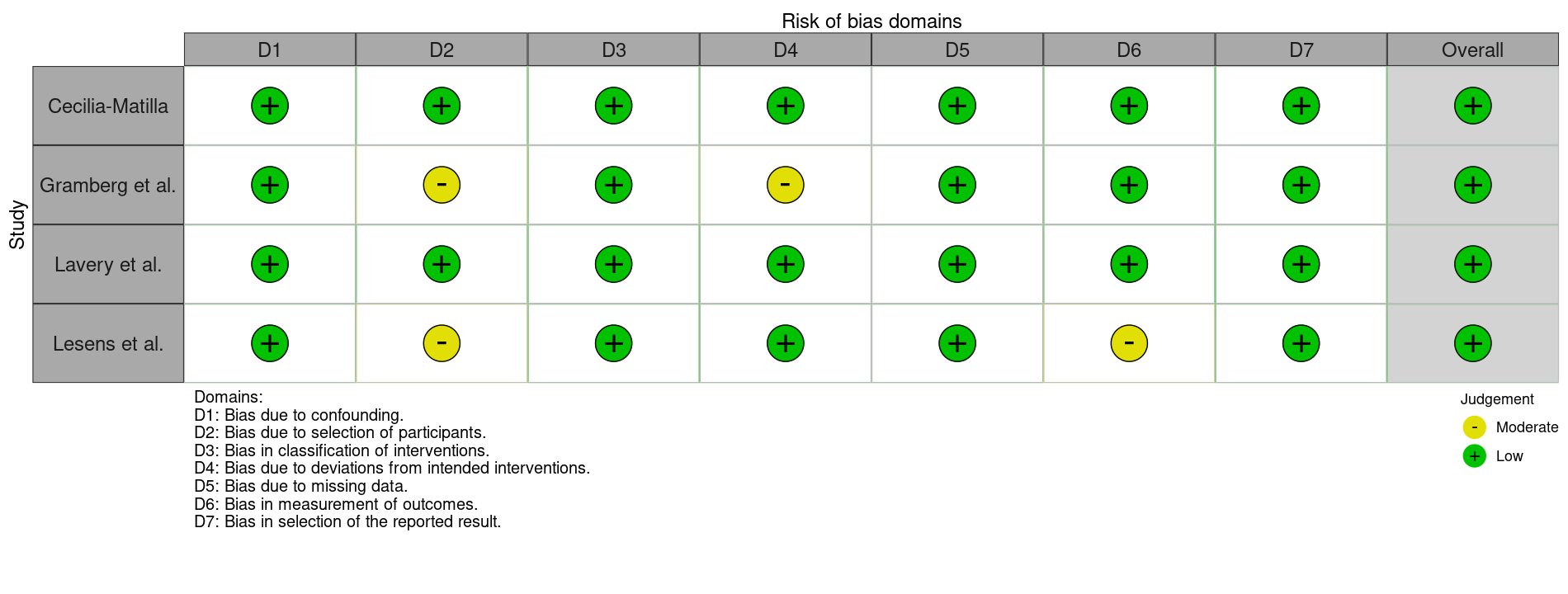


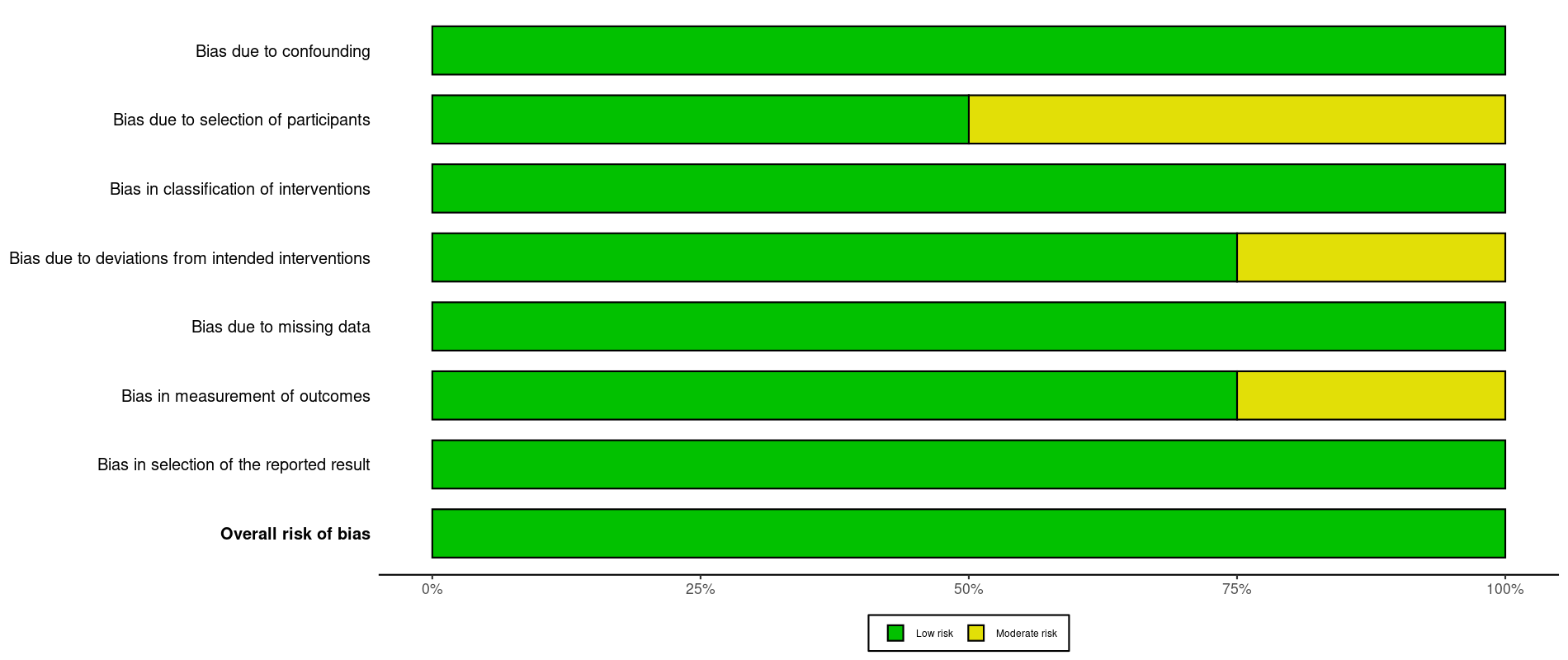


**Supplementary 3: Funnel Plot and Eggers Test**

Eggers Test: p= 0.0684
